# Association between gut microbiota and adrenal disease: a two-sample Mendelian randomized study

**DOI:** 10.3389/fcimb.2024.1421128

**Published:** 2024-07-11

**Authors:** Yue-Yang Zhang, Yao-Wen Liu, Bing-Xue Chen, Qin Wan

**Affiliations:** ^1^ Department of Endocrinology and Metabolism, Affiliated Hospital of Southwest Medical University, Luzhou, China; ^2^ Metabolic Vascular Disease Key Laboratory of Sichuan Province, Luzhou, China; ^3^ Sichuan Clinical Research Center for Diabetes and Metabolism, Luzhou, China; ^4^ Sichuan Clinical Research Center for Nephropathy, Luzhou, China; ^5^ Cardiovascular and Metabolic Diseases Key Laboratory of Luzhou, Luzhou, China; ^6^ Department of Radiation Oncology, Affiliated Hospital of Southwest Medical University, Luzhou, China; ^7^ Department of Ultrasound Medicine, Affiliated Hospital of Southwest Medical University, Luzhou, China

**Keywords:** gut microbiota, adrenal disease, Mendelian randomized, EPI - epidemiology, risk

## Abstract

**Background:**

Some observational studies and clinical experiments suggest a close association between gut microbiota and metabolic diseases. However, the causal effects of gut microbiota on adrenal diseases, including Adrenocortical insufficiency, Cushing syndrome, and Hyperaldosteronism, remain unclear.

**Methods:**

This study conducted a two-sample Mendelian randomization analysis using summary statistics data of gut microbiota from a large-scale genome-wide association study conducted by the MiBioGen Consortium. Summary statistics data for the three adrenal diseases were obtained from the FinnGen study. The study employed Inverse variance weighting, MR-Egger, and MR-PRESSO methods to assess the causal relationship between gut microbiota and these three adrenal diseases. Additionally, a reverse Mendelian randomization analysis was performed for bacteria found to have a causal relationship with these three adrenal diseases in the forward Mendelian randomization analysis. Cochran’s Q statistic was used to test for heterogeneity of instrumental variables.

**Results:**

The IVW test results demonstrate that class Deltaproteobacteria, Family Desulfovibrionaceae, and Order Desulfovibrionales exhibit protective effects against adrenocortical insufficiency. Conversely, Family Porphyromonadaceae, Genus Lachnoclostridium, and Order MollicutesRF9 are associated with an increased risk of adrenocortical insufficiency. Additionally, Family Acidaminococcaceae confers a certain level of protection against Cushing syndrome. In contrast, Class Methanobacteria, Family Lactobacillaceae, Family Methanobacteriaceae, Genus. Lactobacillus and Order Methanobacteriales are protective against Hyperaldosteronism. Conversely, Genus Parasutterella, Genus Peptococcus, and Genus Veillonella are identified as risk factors for Hyperaldosteronism.

**Conclusions:**

This two-sample Mendelian randomization analysis revealed a causal relationship between microbial taxa such as Deltaproteobacteria and Desulfovibrionaceae and Adrenocortical insufficiency, Cushing syndrome, and Hyperaldosteronism. These findings offer new avenues for comprehending the development of adrenal diseases mediated by gut microbiota.

## Introduction

Adrenal diseases are vital components of endocrine system disorders, chiefly encompassing Adrenocortical insufficiency (AI), Cushing syndrome(CS), and Hyperaldosteronism(HA) (Salman and Cohen, 2021). AI manifests as adrenal cortex dysfunction, resulting in absolute or relative insufficiency of cortisol secretion (Bancos et al., 2015). Primary adrenal insufficiency, such as Addison’s disease, is relatively rare and typically arises from direct adrenal failure (Erichsen et al., 2009; Ross and Levitt, 2013; Bornstein et al., 2016). Conversely, secondary adrenal cortex insufficiency, more prevalent, stems mainly from pituitary damage affecting adrenocortical hormone secretion (Grossman, 2010; Hahner et al., 2021). AI frequently progresses to adrenal crisis, significantly heightening patient mortality rates (Dineen et al., 2019). Prolonged elevation of endogenous cortisol levels can result in Cushing’s syndrome, leading to numerous organ complications such as hypertension, obesity, dysregulation of glucose and lipid metabolism, and cognitive impairment (Pivonello et al., 2016). These complications arise due to the impact on the nervous and immune systems, ultimately diminishing the patient’s quality of life (Hatipoglu, 2012; Ferriere and Tabarin, 2020). Hyperaldosteronism primarily involves primary aldosterone elevation and stands as a frequent cause of hypertension, often inflicting direct damage on target organs (Zennaro et al., 2020). Compared to diabetes, the incidence of adrenal diseases is relatively lower. However, with advancements in medical technology, their global incidence is on the rise. The prevalence of CS is 39.1 per million, with an annual incidence of 2.4 per 100,000. Reports indicate that since 1974, the prevalence of CS has increased almost linearly (Steffensen et al., 2010), while the prevalence of AI has reached 100-140 per million (Hahner et al., 2021). HS most commonly manifests as hypertension, and it is estimated that approximately 6%-10% of hypertensive patients are affected by HS (Monticone et al., 2017). These data suggest that adrenal diseases are impacting an increasing number of patients, gradually becoming a significant focus of global public health efforts.

The gut microbiota, defined as the microbial community residing in the human gastrointestinal tract, has garnered increasing attention due to mounting evidence suggesting its close association with various diseases in the body. For example, a case-control study observed a reduction in Bacteroidetes and an increase in Firmicutes and Proteobacteria in patients with CS (Zhang et al., 2022). In a study involving 54 psoriasis patients and 27 healthy controls, genetic material from gut bacteria was detected in the plasma of 16 psoriasis patients, but not in any healthy controls. This discrepancy may be attributed to a reduction in the abundance of potential probiotics in psoriasis patients, resulting in immune system imbalance (Mahmud et al). Moreover, the relationship between obesity and gut microbiota is well established (Gomes et al., 2018). It is widely believed that gut microbiota dysbiosis primarily induces disease by influencing the gut-brain axis and regulating brain function (Anand et al., 2022). Animal studies have shown that microbial colonization in mice affects the development of the hypothalamic-pituitary-adrenal axis postnatally, suggesting that gut microbiota significantly impact adrenal function (Sudo et al., 2004). Another study found that the excessive release of lipopolysaccharides into the blood by Gram-negative bacteria can hyperactivate the hypothalamic-pituitary-adrenal axis, inducing systemic and neuroinflammation, leading to increased cortisol secretion and severely disrupting central nervous system homeostasis (Moylan et al., 2014). The probiotic B. pseudocatenulatum (CECT 7765) has been shown to reverse abnormal stress responses caused by dysregulation of glucocorticoid receptors (Agusti et al., 2018). Treatment with Lactobacillus sp. during early maternal separation stress can normalize hypothalamic-pituitary-adrenal axis activity (Gareau et al., 2007). L. farciminis has also been found to prevent excessive activation of the hypothalamic-pituitary-adrenal axis caused by restraint stress (Ait-Belgnaoui et al., 2012).However, due to challenges in confirming exposure and outcome times in case-control studies, and because similar observational studies draw conclusions based on changes in microbial composition in patients’ feces, they are susceptible to various confounding factors such as age and environment (Rinninella et al., 2019). These limitations impede causal inferences between gut microbiota and adrenal diseases, leaving the causal relationship between gut microbiota and adrenal diseases unresolved.

Mendelian randomization(MR) is currently recognized as a method capable of inferring causality between exposure and outcome. It utilizes genetic variants associated with exposure as instrumental variables to assess the association between exposure and outcome (Greenland, 2000; Emdin et al., 2017). According to Mendelian genetic laws, genetic information is randomly allocated at conception, occurring prior to the onset of any disease. This randomization significantly minimizes the influence of environmental and lifestyle confounding factors. MR has been widely utilized to explore causal relationships between gut microbiota and various diseases, including preeclampsia (Li et al., 2022), autoimmune diseases (Xu et al., 2021), and others. However, research on the relationship between gut microbiota and AI, CS, and HA is relatively scarce. Thus, this study aimed to investigate the causal relationship between gut microbiota and various adrenal diseases by conducting a comprehensive two-sample MR analysis on three adrenal diseases, namely AI, CS, and HA.

## Method

This Mendelian randomization analysis utilized summary-level MR analysis genome-wide association studies(GWAS) data, all of which were publicly available and did not involve the collection of new data, hence no additional ethical approval was required. The study workflow is depicted in **Supplementary Figure 1**.

### Exposure data

The instrumental variables (IVs) for gut microbiota were derived from a large-scale GWAS on human gut microbiota composition conducted by the MiBioGen Consortium (Kurilshikov et al., 2021). This study pooled 16S rRNA sequencing data from a total of 18,340 participants across 24 cohort studies to explore potential associations between common genetic variants and gut microbiota. A total of 211 taxonomic groups (131 genera, 35 families, 20 orders, 16 classes, and 9 phyla) were included in this study.

### Outcome data

GWAS data for the three adrenal diseases were sourced from the FinnGen study, a nationwide GWAS conducted in Finland (Kurki et al., 2023).

### Selection of instrumental variables

In this study, the following criteria were used to select IVs: 1. Single nucleotide polymorphisms(SNPs) significantly associated with gut microbiota were chosen as potential IVs, with a P-value less than the genome-wide significance threshold (5×10e-8); 2. SNPs with strong linkage disequilibrium were excluded based on an R2<0.001, with a clumping window size of 10,000 kb; 3. SNPs with a minor allele frequency ≤ 0.01 were eliminated; 4. In the presence of palindromic SNPs, allele frequency information was used to infer the alleles on the forward strand.

### MR analysis

This study primarily utilized inverse variance-weighted (IVW), MR-Egger regression, and MR-PRESSO methods to examine the causal relationships and horizontal pleiotropy between gut microbiota and three adrenal diseases, with the IVW method being the main approach. Cochran’s IVW Q statistic was used to test the heterogeneity of IVs. Additionally, to identify potential heterogeneity SNPs, a “leave-one-out” analysis was conducted by sequentially excluding each instrumental SNP. Furthermore, reverse MR analysis was performed on gut microbiota identified in forward MR analysis to have causal relationships with the three adrenal diseases, using the same methods as forward MR. To assess the strength of selected SNPs, the following formula was used to calculate the F-statistic for each bacterial taxonomic group:


$$F=\frac{R^{2}(N-1-K)}{\left(1-R^{2}\right)K}$$

where R^2^ represents the proportion of exposure variance explained by genetic variation, N represents the sample size, and K represents the number of IVs. If the corresponding F-statistic is >10, then no significant weak instrument bias is considered (Pierce et al., 2011).

All statistical analyses were conducted using R version 4.3.3 (R Foundation for Statistical Computing). The TwosampleMR (version 0.5.11) packages were used for MR analysis.

## Result

### SNP selection

According to the criteria for selecting IVs, we respectively used 81, 8, and 99 SNPs as IVs for AI, CS, and HA. The F-statistics for all selected IVs were >10 (detailed in **Supplementary Table 1**), indicating the exclusion of weak instrument bias as much as possible. The results of Cochran’s IVW Q test and MR-PRESSO global test showed no significant heterogeneity among these IVs (**Supplementary Table 3**, **Supplementary Table 5**). Furthermore, the MR-Egger regression intercept analysis showed no significant horizontal pleiotropy.

### Causal influence of gut microbiota on the development of three adrenal diseases

#### Adrenocortical insufficiency

**Figure 1:**
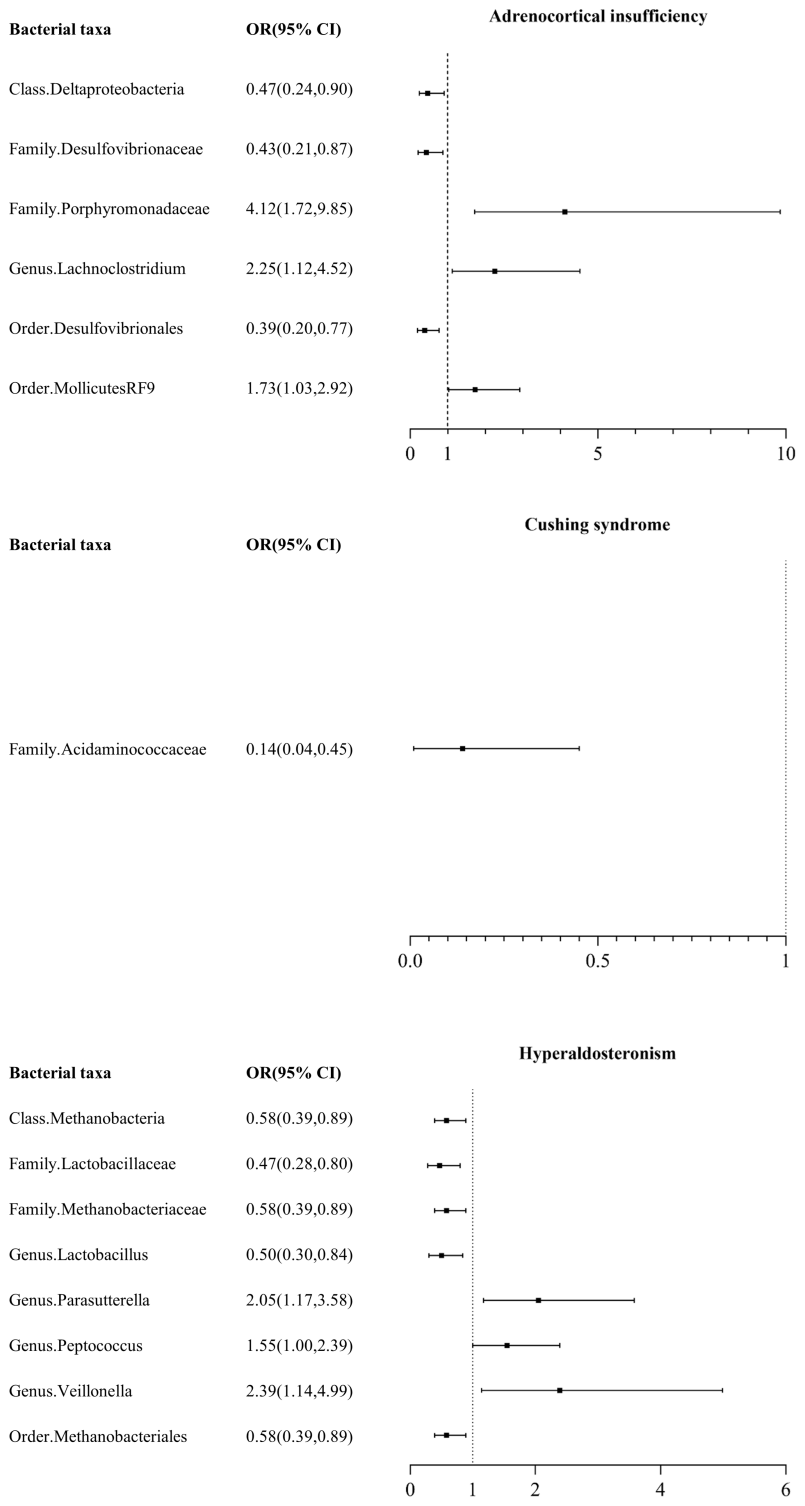
Mendelian randomization results of causal effects between gut microbiome and adrenal disease.

Among the 211 taxa of gut microbiota, we identified 6 bacterial taxa associated with AI, namely class Deltaproteobacteria, Family Desulfovibrionaceae, Family Porphyromonadaceae, Genus Lachnoclostridium, Order Desulfovibrionales, and Order MollicutesRF9. IVW test results showed that class Deltaproteobacteria, Family Desulfovibrionaceae, and Order Desulfovibrionales were protective factors for AI, with odds ratios (ORs) of 0.47 (95% CI: 0.24, 0.90), 0.43 (95% CI: 0.21, 0.87), and 0.39 (95% CI: 0.20, 0.77), respectively, while Family Porphyromonadaceae, Genus Lachnoclostridium, and Order MollicutesRF9 were risk factors for AI, with ORs of 4.12 (95% CI: 1.72, 9.85), 2.25 (95% CI: 1.12, 4.52), and 1.73 (95% CI: 1.03, 2.92), respectively (**Figure 1**, **Supplementary Table 2**).

#### Cushing syndrome

Among the 211 taxa of gut microbiota, we found that only Family Acidaminococcaceae exhibited a certain protective effect against CS, with an odds ratio (OR) of 0.14 (95% CI: 0.04, 0.45) (**Figure 1**, **Supplementary Table 2**).

#### Hyperaldosteronism

Among the 211 taxa of gut microbiota, we found that Class Methanobacteria (OR: 0.58, 95% CI: 0.39, 0.89), Family Lactobacillaceae (OR: 0.47, 95% CI: 0.28, 0.80), Family Methanobacteriaceae (OR: 0.58, 95% CI: 0.39, 0.89), Genus Lactobacillus (OR: 0.50, 95% CI: 0.30, 0.84), and Order Methanobacteriales (OR: 0.58, 95% CI: 0.39, 0.89) were protective factors for HA, while Genus Parasutterella (OR: 2.05, 95% CI: 1.17, 3.58), Genus Peptococcus (OR: 1.55, 95% CI: 1.00, 2.39), and Genus Veillonella (OR: 2.39, 95% CI: 1.14, 4.99) were risk factors for HA.

### Sensitivity analyses

**Figure 2:**
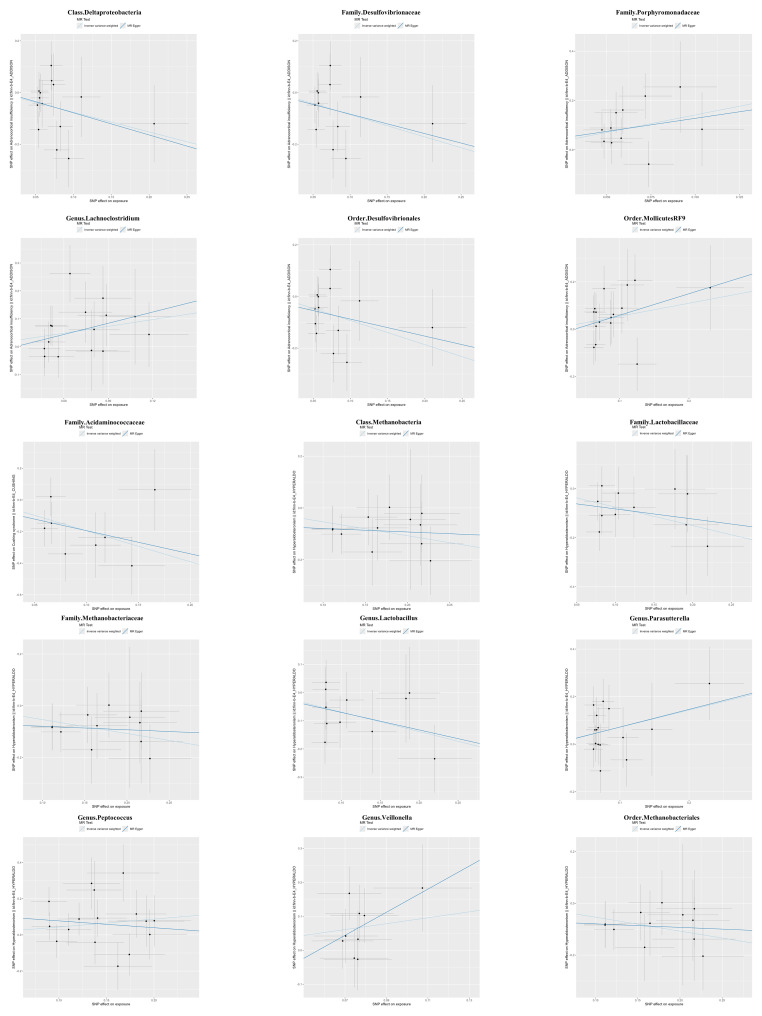
Scatterplots of the causal relationship between gut microbiota and adrenal diseases.

**Figure 3:**
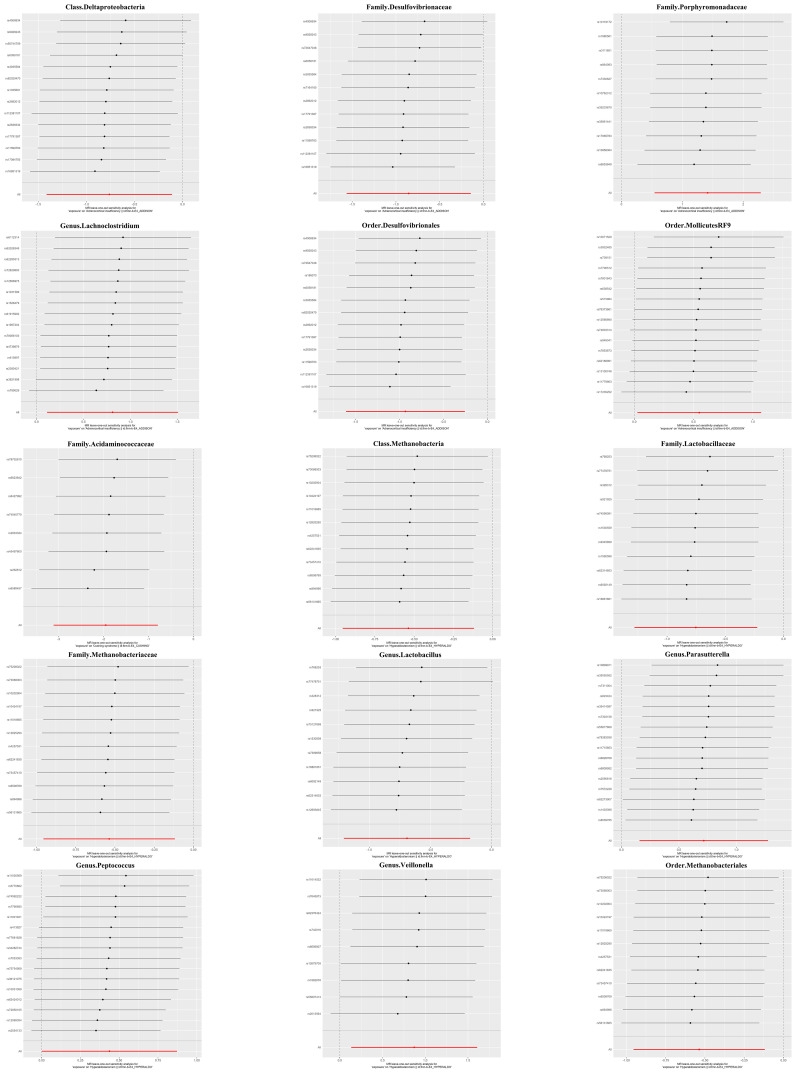
Leave-one-out plots for the causal association between gut microbiota and adrenal diseases.

The magnitude and direction of causal estimates obtained by the MR-Egger method were similar to those of the IVW method (**Figure 2** and **Supplementary Table 2**). In the scatter plot (**Figure 2**) and leave-one-out plot (**Figure 3**), potential outliers were visually observed for some bacterial taxa IVs. Still, further, Cochran’s IVW Q test results and MR-PRESSO global test results showed no significant heterogeneity (**Supplementary Table 3**, **Supplementary Table 5**). Additionally, when using the MR-Egger regression intercept method, we found no evidence of horizontal pleiotropy in the causal relationship between the three adrenal diseases and gut microbiota (**Supplementary Table 4**).

### Reverse causality between 3 adrenal diseases and gut microbiota

According to the results of reverse MR analysis, we did not find significant causal relationships between the three adrenal diseases and corresponding gut microbiota, and MR-Egger regression intercept analysis also did not reveal significant horizontal pleiotropy (**Supplementary Table 6**).

## Discussion

In this study, we conducted a two-sample MR analysis using summary statistics data on human gut microbiota from the large-scale GWAS analysis conducted by the MiBioGen consortium and summary statistics data on three adrenal diseases from the FinnGen study. The aim was to evaluate the causal relationships between gut microbiota and the three adrenal diseases. Our findings revealed that Class Deltaproteobacteria, Family Desulfovibrionaceae, and Order Desulfovibrionales were protective factors for AI, while Family Acidaminococcaceae exhibited a protective effect against CS. Additionally, Class Methanobacteria, Family Lactobacillaceae, Family Methanobacteriaceae, Genus Lactobacillus, and Order Methanobacteriales were identified as protective factors for HA.

Increasing research indicates a causal relationship between the gut microbiota we selected and various diseases. For instance, studies have identified Class Deltaproteobacteria as a major risk factor for Graves’ disease and a potential risk factor for chronic kidney disease (Cao et al., 2023; Luo et al., 2023). Meanwhile, Desulfovibrionaceae is recognized as a harmful bacterial genus in the gut, capable of producing toxic effects on the intestinal epithelium by reducing sulfate to H2S under anaerobic conditions, leading to gastrointestinal diseases (Cabrera et al., 2006; Pires et al., 2006). Some research has found a significant correlation between high concentrations of Desulfovibrionaceae and the development of Parkinson’s disease (Murros et al., 2021). However, a study from the Guangdong Gut Microbiome Program in China revealed a negative correlation between the relative abundance of Desulfovibrionaceae and BMI, waist circumference, and uric acid levels (Chen et al., 2021). Our study also discovered a protective effect of Desulfovibrionaceae against AI, suggesting that Desulfovibrionaceae is not universally associated with adverse health conditions. This could be attributed to the positive correlation between the relative abundance of Desulfovibrionaceae and microbial diversity, which benefits the stability of the microbiome and host health (Le Chatelier et al., 2013).

Some studies suggest that Porphyromonadaceae may act as regulators of obesity by producing short-chain fatty acids such as acetate and propionate (Lu et al., 2016). However, research by Teresa Tavella (Tavella et al., 2021) and others found that elderly individuals with higher concentrations of Porphyromonadaceae have significantly lower serum levels of branched-chain amino acids, which are associated with insulin deficiency and insulin resistance (Rietman et al., 2016; Holeček, 2018). This may also be a reason for the increased risk of Adrenocortical insufficiency (AI). Lachnoclostridium is one of the core genera in the gut microbiota and is significantly associated with many metabolic diseases. Some studies suggest that a high abundance of Lachnoclostridium may decrease levels of acetate in circulation, leading to increased abdominal fat and negative effects on obesity and type 2 diabetes (Cai et al., 2022).

There is currently limited research on MollicutesRF9, but we found that it increases the risk of AI. We speculate that this may be similar to mycoplasma, which also belongs to Mollicutes, but the specific mechanism needs further exploration.

Acidaminococcaceae mainly produces butyrate in the human body. Clinical evidence suggests that the abundance of bacteria-producing butyrate is associated with blood pressure reduction in obese pregnant women. A recent study found that supplementing fiber and acetate can improve gut dysbiosis, related to the increase of Acidaminococcaceae. Acidaminococcaceae may play a protective role in hypertension and heart failure in hypertensive mice (Xu et al., 2020).

Methanobacteria are mainly located in the human gastrointestinal tract and are responsible for methane production (Hoegenauer et al., 2022). Compared to healthy individuals, patients with inflammatory bowel disease, periodontal disease (Lepp et al., 2004), obesity (Maya-Lucas et al., 2019), and cancer (Cai et al., 2022) have higher concentrations of Methanobacteria, but there is currently no evidence to suggest that they are pathogens (Mafra et al., 2022). Lactobacillaceae, as a well-recognized probiotic, is also the most widely used microbial genus (Gourbeyre et al., 2011). It can protect the body by enhancing the intestinal epithelial barrier (Collado et al., 2005), producing antimicrobial substances (Bierbaum and Sahl, 2009), and playing an immunomodulatory role (Lebeer et al., 2010). Research on Parasutterella is currently limited. Some studies have found that an increase in the abundance of Parasutterella is associated with a decrease in gut microbiota diversity (Chiodini et al., 2015) and gut dysbiosis may be associated with many metabolic diseases (Carding et al., 2015; Chen et al., 2018). This study found that the concentration of Parasutterella is elevated in patients with HA, but the specific mechanism is unclear and requires further exploration through more basic research.

This study has several strengths. It determined the causal relationship between gut microbiota and three adrenal diseases through MR analysis, minimizing the interference of confounding factors, and supplemented by reverse MR analysis. The genetic variation of gut microbiota was obtained from the largest GWAS meta-analysis, ensuring the strength of the instruments in MR analysis. Horizontal pleiotropy was detected and eliminated using MR-PRESSO and MR-Egger regression intercept tests. A two-sample MR design was used, and non-overlapping summary-level data for exposure and outcome were used to avoid bias (Burgess et al., 2016).

However, this study also has some limitations. Firstly, because the data used in this study are summary-level GWAS data, subgroup analysis cannot be performed. Secondly, most patients in this study are of European descent, so the results may not apply to other ethnic groups. Thirdly, due to the strict criteria used in the selection of IVs in this study, a large number of SNPs of gut microbiota were excluded, so false discovery rate (FDR) correction was not applied to the research results, which may introduce some errors. Finally, we must acknowledge the inherent limitations of MR, including trait heterogeneity and developmental compensation, which may impact the accuracy and applicability of our research findings (Haycock et al., 2016).

## Conclusions

In conclusion, we comprehensively evaluated the causal relationship between gut microbiota and three adrenal diseases. Our results indicate that microbial taxa such as Deltaproteobacteria and Desulfovibrionaceae may serve as pathways for the diagnosis and treatment of AI, CS, and HA, which require further experimental exploration. This study may provide new directions for understanding the occurrence and development of adrenal diseases mediated by gut microbiota.

## Data availability statement

The original contributions presented in the study are included in the article/**Supplementary Material**. Further inquiries can be directed to the corresponding author.

## Author contributions

Y-YZ: Conceptualization, Formal analysis, Methodology, Writing – original draft. Y-WL: Data curation, Methodology, Writing – original draft. B-XC: Software, Visualization, Writing – original draft. QW: Funding acquisition, Validation, Writing – review & editing.
