# Supplementary material for: Association between gut microbiota and adrenal disease: a two-sample Mendelian randomized study
Source: Front Cell Infect Microbiol. 2024 Jul 11;14:1421128. doi: 10.3389/fcimb.2024.1421128 (PMC11269257; doi:10.3389/fcimb.2024.1421128)
Supplement: Supplementary file 1 [file DataSheet_1.docx]

**Supplementary material**

**Figure and tables of contents**

[Figure S1. Study design and workflow 2](#_Toc162624227)

[Table S1 Instrumental variables used in MR analysis of the association between gut microbiota and adrenal disease 3](#_Toc162624229)

[Table S2 Full result of MR estimates for the association between gut microbiota and adrenal disease 10](#_Toc162624230)

[Table S3 The heterogeneity of gut microbiota instrumental variables. 12](#_Toc162624231)

[Table S4 Directional horizontal pleiotropy assessed by intercept term in MR Egger regression of the association between gut microbiota and adrenal disease 14](#_Toc162624232)

[Table S5 MR-PRESSO analysis for the association between gut microbiota and adrenal disease 15](#_Toc162624233)

[Table S6 Full result of MR estimates for the association between adrenal disease and gut microbiota 16](#_Toc162624234)

Figure S1. Study design and workflow


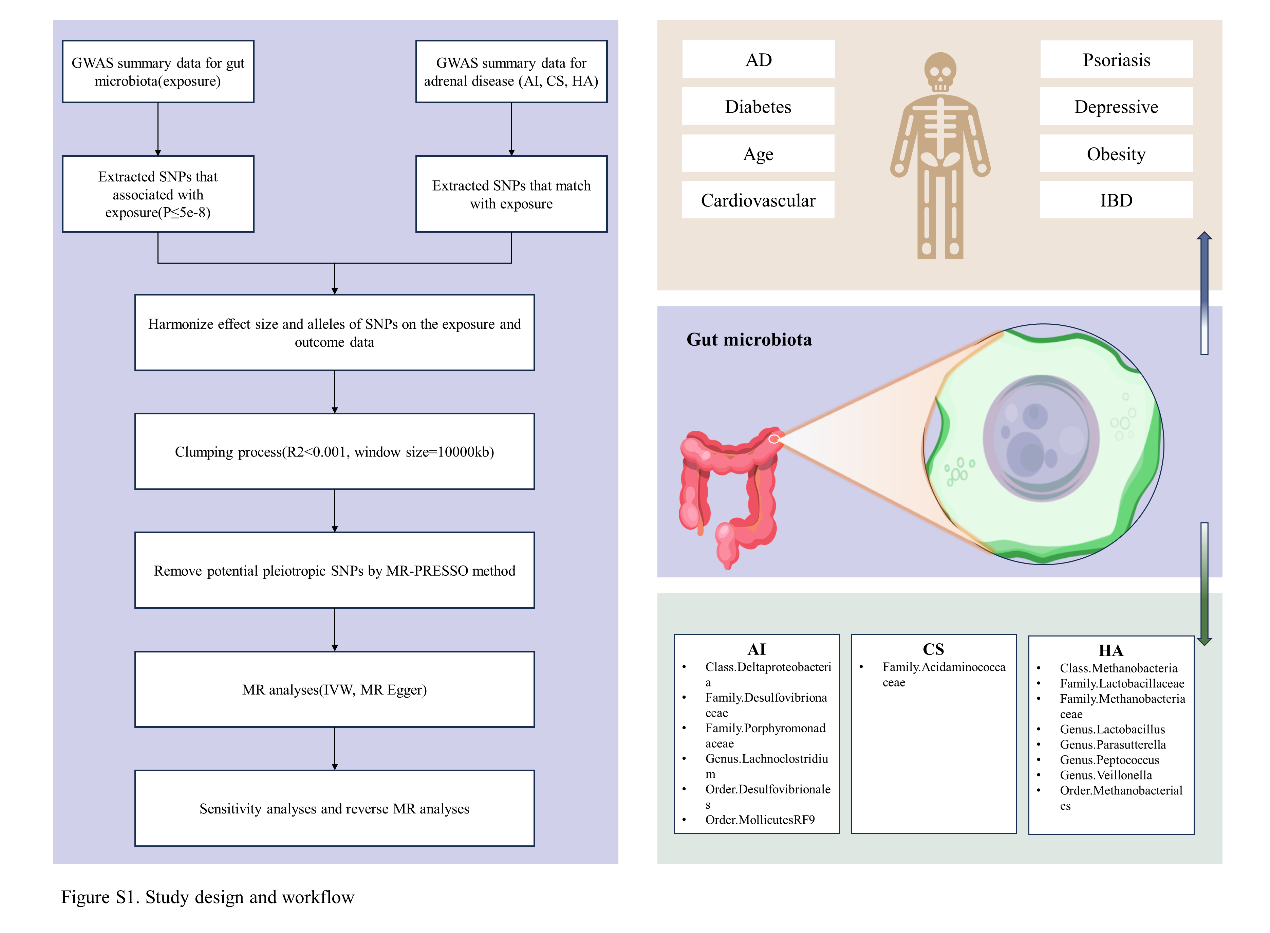


Table S1 Instrumental variables used in MR analysis of the association between gut microbiota and adrenal disease

| Bacterial taxa (exposure) | SNP | F-statistic | Effect allele | Other allele | Exposure (Bacteria) | | | Outcome (adrenal disease) | | |
| --- | --- | --- | --- | --- | --- | --- | --- | --- | --- | --- |
|  |  |  |  |  | Beta | SE | P-value | Beta | SE | P-value |
| *Adrenocortical insufficiency* | | | | | | | | | | |
| Class. Deltaproteobacteria | rs1035691 | 20.59 | A | G | -0.055 | 0.012 | 9.65e-06 | 0.021 | 0.073 | 0.777 |
|  | rs112381107 | 20.44 | C | T | 0.207 | 0.046 | 4.63e-06 | -0.120 | 0.149 | 0.419 |
|  | rs11599763 | 21.49 | T | C | -0.054 | 0.012 | 3.94e-06 | -0.006 | 0.073 | 0.934 |
|  | rs16851319 | 21.87 | G | C | -0.071 | 0.015 | 5.68e-06 | -0.105 | 0.094 | 0.265 |
|  | rs17084793 | 19.85 | G | A | -0.071 | 0.016 | 5.69e-06 | -0.046 | 0.101 | 0.649 |
|  | rs17791387 | 22.76 | A | G | -0.074 | 0.015 | 1.6e-06 | -0.031 | 0.121 | 0.799 |
|  | rs2692012 | 18.97 | A | G | 0.110 | 0.025 | 3.14e-06 | -0.017 | 0.155 | 0.913 |
|  | rs2838334 | 20.48 | G | A | 0.056 | 0.012 | 5.45e-06 | -0.001 | 0.074 | 0.992 |
|  | rs3935584 | 20.48 | C | T | -0.052 | 0.012 | 7.5e-06 | 0.048 | 0.071 | 0.496 |
|  | rs4506934 | 21.66 | C | T | -0.094 | 0.020 | 3.59e-06 | 0.255 | 0.111 | 0.021 |
|  | rs55744759 | 20.85 | A | G | -0.078 | 0.017 | 7.31e-06 | 0.222 | 0.112 | 0.048 |
|  | rs6058181 | 24.75 | C | T | 0.083 | 0.017 | 3.4e-07 | -0.131 | 0.096 | 0.172 |
|  | rs62020470 | 20.48 | A | G | -0.059 | 0.013 | 4.85e-06 | 0.043 | 0.092 | 0.642 |
|  | rs9928243 | 20.92 | C | A | -0.054 | 0.012 | 5.02e-06 | 0.143 | 0.071 | 0.044 |
| Family. Desulfovibrionaceae | rs112381107 | 21.40 | C | T | 0.211 | 0.046 | 2.82e-06 | -0.120 | 0.149 | 0.419 |
|  | rs11599763 | 22.38 | T | C | -0.056 | 0.012 | 2.5e-06 | -0.006 | 0.073 | 0.934 |
|  | rs16851319 | 23.55 | G | C | -0.073 | 0.015 | 2.4e-06 | -0.105 | 0.094 | 0.265 |
|  | rs17791387 | 22.31 | A | G | -0.073 | 0.015 | 2.1e-06 | -0.031 | 0.121 | 0.799 |
|  | rs2692012 | 20.29 | A | G | 0.114 | 0.025 | 1.56e-06 | -0.017 | 0.155 | 0.913 |
|  | rs2838334 | 21.15 | G | A | 0.057 | 0.012 | 3.82e-06 | -0.001 | 0.074 | 0.992 |
|  | rs3935584 | 20.63 | C | T | -0.053 | 0.012 | 6.78e-06 | 0.048 | 0.071 | 0.496 |
|  | rs4506934 | 21.94 | C | T | -0.094 | 0.020 | 3.16e-06 | 0.255 | 0.111 | 0.021 |
|  | rs6058181 | 25.25 | C | T | 0.083 | 0.017 | 2.7e-07 | -0.131 | 0.096 | 0.172 |
|  | rs7164160 | 19.68 | A | T | -0.057 | 0.013 | 7.34e-06 | 0.041 | 0.092 | 0.655 |
|  | rs72647048 | 20.30 | T | C | -0.077 | 0.017 | 9.61e-06 | 0.221 | 0.112 | 0.049 |
|  | rs9928243 | 21.14 | C | A | -0.054 | 0.012 | 4.48e-06 | 0.143 | 0.071 | 0.044 |
| Family. Porphyromonadaceae | rs10119172 | 26.74 | C | G | -0.074 | 0.014 | 1.32e-07 | 0.058 | 0.093 | 0.533 |
|  | rs10762312 | 19.42 | G | A | -0.052 | 0.012 | 8.7e-06 | -0.089 | 0.076 | 0.246 |
|  | rs10858364 | 20.95 | G | T | 0.055 | 0.012 | 4.31e-06 | 0.151 | 0.084 | 0.072 |
|  | rs17065783 | 23.40 | A | G | -0.059 | 0.012 | 1.79e-06 | -0.162 | 0.097 | 0.096 |
|  | rs1980561 | 19.72 | A | G | -0.049 | 0.011 | 8.95e-06 | -0.035 | 0.071 | 0.626 |
|  | rs3111851 | 23.07 | C | G | 0.058 | 0.012 | 2.19e-06 | 0.047 | 0.081 | 0.559 |
|  | rs35233670 | 19.95 | T | C | -0.047 | 0.011 | 7.91e-06 | -0.081 | 0.071 | 0.252 |
|  | rs35961441 | 19.49 | A | C | 0.092 | 0.021 | 8.37e-06 | 0.255 | 0.185 | 0.169 |
|  | rs6953849 | 22.70 | A | G | 0.072 | 0.015 | 2.44e-06 | 0.218 | 0.092 | 0.018 |
|  | rs7330827 | 19.14 | T | C | -0.104 | 0.024 | 8.05e-06 | -0.083 | 0.150 | 0.578 |
|  | rs864093 | 20.19 | A | C | -0.053 | 0.012 | 9.6e-06 | -0.029 | 0.087 | 0.740 |
| Genus. Lachnoclostridium | rs1031599 | 20.04 | G | T | -0.079 | 0.018 | 6.31e-06 | 0.013 | 0.145 | 0.926 |
|  | rs12566975 | 19.58 | T | C | -0.047 | 0.011 | 9.57e-06 | 0.007 | 0.071 | 0.925 |
|  | rs1528479 | 19.78 | G | A | -0.050 | 0.011 | 9.64e-06 | -0.017 | 0.073 | 0.815 |
|  | rs1997204 | 19.94 | T | C | -0.108 | 0.024 | 5.97e-06 | -0.108 | 0.172 | 0.530 |
|  | rs2385421 | 17.04 | A | G | 0.075 | 0.018 | 7.14e-06 | 0.124 | 0.110 | 0.261 |
|  | rs3821998 | 20.14 | C | A | -0.086 | 0.019 | 6.72e-06 | -0.174 | 0.115 | 0.131 |
|  | rs4738679 | 20.81 | G | A | -0.052 | 0.011 | 4.42e-06 | -0.074 | 0.073 | 0.310 |
|  | rs6112314 | 26.96 | A | C | -0.056 | 0.011 | 2.43e-07 | 0.036 | 0.075 | 0.632 |
|  | rs615997 | 23.09 | T | C | 0.051 | 0.011 | 2.03e-06 | 0.076 | 0.070 | 0.280 |
|  | rs61915992 | 21.79 | A | T | 0.080 | 0.017 | 2.67e-06 | 0.062 | 0.101 | 0.540 |
|  | rs62028349 | 19.67 | G | C | 0.047 | 0.011 | 9.17e-06 | -0.035 | 0.071 | 0.623 |
|  | rs62285313 | 22.65 | A | G | 0.086 | 0.018 | 1.58e-06 | -0.016 | 0.119 | 0.893 |
|  | rs72829893 | 19.20 | G | T | 0.117 | 0.027 | 5.58e-06 | 0.044 | 0.116 | 0.707 |
|  | rs78068103 | 20.81 | A | G | 0.089 | 0.019 | 3.67e-06 | 0.113 | 0.111 | 0.310 |
|  | rs789029 | 21.60 | C | T | -0.064 | 0.014 | 3.75e-06 | -0.262 | 0.102 | 0.010 |
| Order. Desulfovibrionales | rs112381107 | 21.13 | C | T | 0.210 | 0.046 | 3.22e-06 | -0.120 | 0.149 | 0.419 |
|  | rs11599763 | 22.30 | T | C | -0.055 | 0.012 | 2.61e-06 | -0.006 | 0.073 | 0.934 |
|  | rs16851319 | 23.33 | G | C | -0.073 | 0.015 | 2.7e-06 | -0.105 | 0.094 | 0.265 |
|  | rs17791387 | 22.21 | A | G | -0.073 | 0.015 | 2.25e-06 | -0.031 | 0.121 | 0.799 |
|  | rs186073 | 19.93 | T | C | 0.053 | 0.012 | 8.74e-06 | -0.105 | 0.072 | 0.146 |
|  | rs2692012 | 19.59 | A | G | 0.112 | 0.025 | 2.27e-06 | -0.017 | 0.155 | 0.913 |
|  | rs2838334 | 20.98 | G | A | 0.057 | 0.012 | 4.17e-06 | -0.001 | 0.074 | 0.992 |
|  | rs3935584 | 20.53 | C | T | -0.052 | 0.012 | 7.2e-06 | 0.048 | 0.071 | 0.496 |
|  | rs4506934 | 22.41 | C | T | -0.095 | 0.020 | 2.43e-06 | 0.255 | 0.111 | 0.021 |
|  | rs6058181 | 25.38 | C | T | 0.084 | 0.017 | 2.53e-07 | -0.131 | 0.096 | 0.172 |
|  | rs62020470 | 19.63 | A | G | -0.057 | 0.013 | 7.51e-06 | 0.043 | 0.092 | 0.642 |
|  | rs72647048 | 20.43 | T | C | -0.077 | 0.017 | 9e-06 | 0.221 | 0.112 | 0.049 |
|  | rs9928243 | 21.38 | C | A | -0.054 | 0.012 | 3.97e-06 | 0.143 | 0.071 | 0.044 |
| Order. MollicutesRF9 | rs10071529 | 20.15 | G | C | 0.125 | 0.028 | 8.64e-06 | -0.147 | 0.113 | 0.193 |
|  | rs11779863 | 20.14 | G | A | -0.077 | 0.017 | 6.69e-06 | -0.171 | 0.098 | 0.079 |
|  | rs12566890 | 18.19 | T | G | -0.103 | 0.024 | 8.11e-06 | -0.089 | 0.106 | 0.401 |
|  | rs13100746 | 20.05 | C | T | 0.064 | 0.014 | 7.29e-06 | 0.087 | 0.071 | 0.222 |
|  | rs17235252 | 22.80 | T | C | -0.122 | 0.026 | 2.16e-06 | -0.207 | 0.110 | 0.060 |
|  | rs3932485 | 19.55 | C | T | 0.063 | 0.014 | 9.93e-06 | -0.077 | 0.072 | 0.288 |
|  | rs515984 | 21.07 | T | C | -0.088 | 0.019 | 6.61e-06 | -0.050 | 0.111 | 0.656 |
|  | rs62188991 | 21.09 | G | C | -0.111 | 0.024 | 5.27e-06 | -0.187 | 0.152 | 0.220 |
|  | rs638542 | 20.20 | G | A | -0.071 | 0.016 | 5.17e-06 | -0.030 | 0.078 | 0.696 |
|  | rs739151 | 21.80 | C | G | 0.065 | 0.014 | 3.09e-06 | -0.065 | 0.071 | 0.357 |
|  | rs74603314 | 22.20 | T | C | 0.231 | 0.049 | 2.28e-06 | 0.175 | 0.180 | 0.329 |
|  | rs76373661 | 20.02 | G | A | 0.091 | 0.020 | 5.16e-06 | 0.063 | 0.096 | 0.517 |
|  | rs7706512 | 22.37 | G | A | 0.066 | 0.014 | 2.27e-06 | 0.013 | 0.071 | 0.859 |
|  | rs7801843 | 19.98 | A | G | -0.087 | 0.019 | 9.47e-06 | -0.026 | 0.099 | 0.791 |
|  | rs7853673 | 19.93 | G | A | -0.062 | 0.014 | 6.73e-06 | -0.073 | 0.071 | 0.305 |
|  | rs949341 | 19.89 | G | A | 0.066 | 0.015 | 7.73e-06 | 0.072 | 0.079 | 0.363 |
| *Cushing syndrome* | | | | | | | | | | |
| Family. Acidaminococcaceae | rs262812 | 21.31 | T | C | -0.066 | 0.014 | 3.25e-06 | -0.020 | 0.122 | 0.867 |
|  | rs2933324 | 22.31 | A | G | -0.066 | 0.014 | 2.24e-06 | 0.147 | 0.133 | 0.270 |
|  | rs45497800 | 20.97 | T | C | -0.118 | 0.026 | 5.86e-06 | 0.237 | 0.161 | 0.139 |
|  | rs6427992 | 21.13 | G | C | -0.060 | 0.013 | 4.24e-06 | 0.179 | 0.115 | 0.121 |
|  | rs6589457 | 22.53 | A | G | 0.166 | 0.035 | 2.32e-06 | 0.064 | 0.257 | 0.802 |
|  | rs6923842 | 22.14 | T | C | -0.080 | 0.017 | 2.21e-06 | 0.341 | 0.174 | 0.049 |
|  | rs74540770 | 20.02 | G | A | -0.109 | 0.024 | 7.09e-06 | 0.286 | 0.207 | 0.167 |
|  | rs78702810 | 19.77 | T | C | -0.144 | 0.032 | 9.16e-06 | 0.416 | 0.184 | 0.024 |
| *Hyperaldosteronism* | | | | | | | | | | |
| Class. Methanobacteria | rs10202904 | 26.75 | T | G | -0.122 | 0.024 | 3.01e-07 | 0.101 | 0.079 | 0.199 |
|  | rs10424197 | 20.20 | G | A | -0.111 | 0.025 | 9.28e-06 | 0.084 | 0.089 | 0.346 |
|  | rs11018665 | 19.79 | A | T | 0.111 | 0.025 | 6.52e-06 | -0.080 | 0.090 | 0.371 |
|  | rs12825290 | 19.28 | C | G | -0.217 | 0.049 | 6.08e-06 | 0.138 | 0.162 | 0.393 |
|  | rs4257531 | 20.30 | G | A | 0.164 | 0.036 | 7.44e-06 | -0.077 | 0.127 | 0.543 |
|  | rs56131665 | 20.66 | G | A | 0.179 | 0.039 | 6.18e-06 | 0.002 | 0.125 | 0.985 |
|  | rs62241835 | 23.67 | G | T | -0.203 | 0.042 | 1.63e-06 | 0.044 | 0.272 | 0.871 |
|  | rs6508769 | 19.84 | T | C | 0.154 | 0.034 | 8.23e-06 | -0.035 | 0.109 | 0.745 |
|  | rs73068003 | 20.20 | G | T | -0.158 | 0.035 | 8.45e-06 | 0.170 | 0.130 | 0.192 |
|  | rs73457410 | 24.30 | A | G | 0.215 | 0.044 | 1.41e-06 | -0.065 | 0.157 | 0.678 |
|  | rs75208022 | 21.70 | C | T | -0.227 | 0.049 | 5.92e-06 | 0.204 | 0.132 | 0.122 |
|  | rs894996 | 23.34 | C | A | 0.217 | 0.045 | 1.88e-06 | -0.021 | 0.150 | 0.888 |
| Family. Lactobacillaceae | rs1530559 | 19.06 | G | A | 0.077 | 0.018 | 9.65e-06 | -0.052 | 0.078 | 0.505 |
|  | rs1590596 | 22.23 | C | G | -0.104 | 0.022 | 3.9e-06 | 0.018 | 0.106 | 0.865 |
|  | rs16861661 | 25.96 | G | A | -0.193 | 0.038 | 2.7e-07 | 0.021 | 0.157 | 0.893 |
|  | rs328312 | 24.06 | T | A | 0.083 | 0.017 | 8.69e-07 | -0.110 | 0.077 | 0.155 |
|  | rs6092149 | 23.50 | A | T | -0.083 | 0.017 | 1.38e-06 | -0.012 | 0.078 | 0.882 |
|  | rs62314653 | 20.37 | C | A | 0.177 | 0.039 | 6.59e-06 | -0.001 | 0.164 | 0.993 |
|  | rs74599091 | 20.31 | A | G | 0.192 | 0.043 | 7.7e-06 | -0.147 | 0.285 | 0.606 |
|  | rs768253 | 21.56 | T | G | -0.079 | 0.017 | 3.61e-06 | 0.177 | 0.077 | 0.023 |
|  | rs77478751 | 21.41 | A | G | -0.219 | 0.047 | 5.96e-06 | 0.235 | 0.121 | 0.051 |
|  | rs921925 | 24.55 | A | C | 0.100 | 0.020 | 5.77e-07 | -0.105 | 0.094 | 0.262 |
|  | rs9345899 | 19.77 | A | G | -0.124 | 0.028 | 9.45e-06 | 0.076 | 0.126 | 0.545 |
| Family. Methanobacteriaceae | rs10202904 | 26.75 | T | G | -0.122 | 0.024 | 3.01e-07 | 0.101 | 0.079 | 0.199 |
|  | rs10424197 | 20.20 | G | A | -0.111 | 0.025 | 9.28e-06 | 0.084 | 0.089 | 0.346 |
|  | rs11018665 | 19.79 | A | T | 0.111 | 0.025 | 6.52e-06 | -0.080 | 0.090 | 0.371 |
|  | rs12825290 | 19.28 | C | G | -0.217 | 0.049 | 6.08e-06 | 0.138 | 0.162 | 0.393 |
|  | rs4257531 | 20.30 | G | A | 0.164 | 0.036 | 7.44e-06 | -0.077 | 0.127 | 0.543 |
|  | rs56131665 | 20.66 | G | A | 0.179 | 0.039 | 6.18e-06 | 0.002 | 0.125 | 0.985 |
|  | rs62241835 | 23.67 | G | T | -0.203 | 0.042 | 1.63e-06 | 0.044 | 0.272 | 0.871 |
|  | rs6508769 | 19.84 | T | C | 0.154 | 0.034 | 8.23e-06 | -0.035 | 0.109 | 0.745 |
|  | rs73068003 | 20.20 | G | T | -0.158 | 0.035 | 8.45e-06 | 0.170 | 0.130 | 0.192 |
|  | rs73457410 | 24.30 | A | G | 0.215 | 0.044 | 1.41e-06 | -0.065 | 0.157 | 0.678 |
|  | rs75208022 | 21.70 | C | T | -0.227 | 0.049 | 5.92e-06 | 0.204 | 0.132 | 0.122 |
|  | rs894996 | 23.34 | C | A | 0.217 | 0.045 | 1.88e-06 | -0.021 | 0.150 | 0.888 |
| Genus. Lactobacillus | rs12693845 | 20.60 | C | T | -0.081 | 0.018 | 8.96e-06 | -0.036 | 0.080 | 0.650 |
|  | rs1530559 | 20.35 | G | A | 0.080 | 0.018 | 4.93e-06 | -0.052 | 0.078 | 0.505 |
|  | rs16861661 | 23.04 | G | A | -0.183 | 0.038 | 1.28e-06 | 0.021 | 0.157 | 0.893 |
|  | rs328312 | 23.13 | T | A | 0.082 | 0.017 | 1.41e-06 | -0.110 | 0.077 | 0.155 |
|  | rs6092149 | 21.83 | A | T | -0.080 | 0.017 | 3.29e-06 | -0.012 | 0.078 | 0.882 |
|  | rs62314653 | 22.62 | C | A | 0.188 | 0.039 | 2.24e-06 | -0.001 | 0.164 | 0.993 |
|  | rs7399658 | 23.31 | G | A | -0.107 | 0.022 | 3.12e-06 | 0.027 | 0.101 | 0.793 |
|  | rs75127669 | 20.27 | C | A | 0.140 | 0.031 | 6.83e-06 | -0.138 | 0.148 | 0.352 |
|  | rs768253 | 21.25 | T | G | -0.079 | 0.017 | 4.25e-06 | 0.177 | 0.077 | 0.023 |
|  | rs77478751 | 21.35 | A | G | -0.220 | 0.048 | 7.33e-06 | 0.235 | 0.121 | 0.051 |
|  | rs921925 | 23.49 | A | C | 0.099 | 0.020 | 9.72e-07 | -0.105 | 0.094 | 0.262 |
| Genus. Parasutterella | rs10899911 | 23.42 | A | G | -0.072 | 0.015 | 1.15e-06 | 0.112 | 0.092 | 0.221 |
|  | rs11715853 | 20.58 | G | A | -0.066 | 0.015 | 6.23e-06 | -0.061 | 0.084 | 0.472 |
|  | rs1403396 | 22.64 | A | T | -0.076 | 0.016 | 2.76e-06 | -0.180 | 0.094 | 0.056 |
|  | rs2090816 | 22.49 | A | C | 0.084 | 0.018 | 2.9e-06 | 0.149 | 0.100 | 0.135 |
|  | rs35055552 | 21.65 | T | C | 0.110 | 0.024 | 3.35e-06 | -0.066 | 0.112 | 0.557 |
|  | rs35414597 | 23.22 | T | A | -0.068 | 0.014 | 1.51e-06 | 0.001 | 0.084 | 0.990 |
|  | rs55877868 | 20.97 | A | C | -0.104 | 0.023 | 2.87e-06 | -0.028 | 0.128 | 0.827 |
|  | rs62273907 | 20.86 | A | G | 0.229 | 0.050 | 5.88e-06 | 0.255 | 0.154 | 0.098 |
|  | rs6809952 | 20.60 | G | A | -0.068 | 0.015 | 8.13e-06 | -0.069 | 0.088 | 0.432 |
|  | rs6828768 | 23.05 | C | T | 0.064 | 0.013 | 1.78e-06 | 0.060 | 0.077 | 0.435 |
|  | rs7303158 | 23.21 | C | T | 0.065 | 0.013 | 1.33e-06 | 0.003 | 0.077 | 0.969 |
|  | rs7311004 | 20.48 | T | C | -0.062 | 0.014 | 5.92e-06 | 0.021 | 0.078 | 0.789 |
|  | rs7572229 | 24.92 | G | A | 0.066 | 0.013 | 6.32e-07 | 0.121 | 0.077 | 0.117 |
|  | rs78383039 | 24.24 | T | C | -0.146 | 0.030 | 1.57e-06 | -0.063 | 0.195 | 0.748 |
|  | rs8039785 | 21.61 | T | G | 0.062 | 0.013 | 3.62e-06 | 0.165 | 0.077 | 0.033 |
|  | rs823424 | 20.66 | G | A | -0.071 | 0.016 | 4.95e-06 | 0.003 | 0.089 | 0.971 |
| Genus. Peptococcus | rs10031059 | 28.77 | T | C | -0.121 | 0.023 | 1.24e-07 | -0.088 | 0.091 | 0.333 |
|  | rs11001941 | 24.86 | G | A | -0.196 | 0.039 | 1.33e-06 | -0.002 | 0.138 | 0.988 |
|  | rs11030569 | 21.63 | A | T | -0.174 | 0.037 | 3.13e-06 | 0.108 | 0.117 | 0.354 |
|  | rs12069354 | 19.50 | C | T | 0.168 | 0.038 | 9.28e-06 | 0.342 | 0.158 | 0.030 |
|  | rs2054133 | 22.60 | G | A | 0.090 | 0.019 | 2.14e-06 | 0.185 | 0.081 | 0.022 |
|  | rs34282744 | 23.00 | G | C | 0.192 | 0.040 | 1.84e-06 | 0.076 | 0.142 | 0.592 |
|  | rs36121075 | 21.09 | A | G | -0.141 | 0.031 | 6.99e-06 | -0.093 | 0.104 | 0.369 |
|  | rs413827 | 21.53 | G | A | 0.110 | 0.024 | 3.3e-06 | 0.030 | 0.090 | 0.740 |
|  | rs5770862 | 20.61 | T | C | 0.162 | 0.036 | 3.22e-06 | -0.173 | 0.133 | 0.194 |
|  | rs62424012 | 22.04 | G | A | 0.137 | 0.029 | 1.15e-06 | 0.248 | 0.160 | 0.121 |
|  | rs7033353 | 22.52 | T | G | 0.090 | 0.019 | 2.22e-06 | 0.047 | 0.077 | 0.539 |
|  | rs72850165 | 19.98 | T | C | -0.134 | 0.030 | 5.74e-06 | -0.285 | 0.144 | 0.048 |
|  | rs74592222 | 20.73 | G | A | 0.138 | 0.030 | 8.55e-06 | -0.041 | 0.122 | 0.737 |
|  | rs75754569 | 32.25 | C | G | 0.181 | 0.032 | 1.1e-08 | 0.116 | 0.132 | 0.381 |
|  | rs7766680 | 20.80 | G | C | 0.098 | 0.021 | 3.51e-06 | -0.035 | 0.092 | 0.701 |
|  | rs77681628 | 26.73 | C | T | 0.200 | 0.039 | 2.69e-07 | 0.078 | 0.141 | 0.579 |
| Genus. Veillonella | rs11614532 | 20.30 | G | C | 0.074 | 0.017 | 7.13e-06 | -0.023 | 0.086 | 0.788 |
|  | rs12679709 | 23.17 | C | G | -0.079 | 0.016 | 1.78e-06 | -0.102 | 0.081 | 0.204 |
|  | rs1882878 | 22.00 | A | G | -0.077 | 0.016 | 2.98e-06 | -0.108 | 0.085 | 0.201 |
|  | rs2013594 | 21.57 | T | C | -0.072 | 0.016 | 3.42e-06 | -0.168 | 0.079 | 0.034 |
|  | rs55807413 | 20.39 | A | G | 0.107 | 0.024 | 5.51e-06 | 0.183 | 0.128 | 0.153 |
|  | rs62376424 | 21.73 | C | T | -0.076 | 0.016 | 3.65e-06 | -0.033 | 0.084 | 0.698 |
|  | rs6656807 | 20.85 | A | G | 0.070 | 0.015 | 5.5e-06 | 0.042 | 0.080 | 0.601 |
|  | rs742016 | 21.13 | A | G | -0.069 | 0.015 | 4.66e-06 | -0.028 | 0.082 | 0.736 |
|  | rs7645873 | 21.49 | A | T | 0.076 | 0.016 | 3.12e-06 | -0.027 | 0.091 | 0.771 |
| Order. Methanobacteriales | rs10202904 | 26.75 | T | G | -0.122 | 0.024 | 3.01e-07 | 0.101 | 0.079 | 0.199 |
|  | rs10424197 | 20.20 | G | A | -0.111 | 0.025 | 9.28e-06 | 0.084 | 0.089 | 0.346 |
|  | rs11018665 | 19.79 | A | T | 0.111 | 0.025 | 6.52e-06 | -0.080 | 0.090 | 0.371 |
|  | rs12825290 | 19.28 | C | G | -0.217 | 0.049 | 6.08e-06 | 0.138 | 0.162 | 0.393 |
|  | rs4257531 | 20.30 | G | A | 0.164 | 0.036 | 7.44e-06 | -0.077 | 0.127 | 0.543 |
|  | rs56131665 | 20.66 | G | A | 0.179 | 0.039 | 6.18e-06 | 0.002 | 0.125 | 0.985 |
|  | rs62241835 | 23.67 | G | T | -0.203 | 0.042 | 1.63e-06 | 0.044 | 0.272 | 0.871 |
|  | rs6508769 | 19.84 | T | C | 0.154 | 0.034 | 8.23e-06 | -0.035 | 0.109 | 0.745 |
|  | rs73068003 | 20.20 | G | T | -0.158 | 0.035 | 8.45e-06 | 0.170 | 0.130 | 0.192 |
|  | rs73457410 | 24.30 | A | G | 0.215 | 0.044 | 1.41e-06 | -0.065 | 0.157 | 0.678 |
|  | rs75208022 | 21.70 | C | T | -0.227 | 0.049 | 5.92e-06 | 0.204 | 0.132 | 0.122 |
|  | rs894996 | 23.34 | C | A | 0.217 | 0.045 | 1.88e-06 | -0.021 | 0.150 | 0.888 |
| MR, Mendelian randomization; SNP, single nucleotide polymorphism; SE, standard error. | | | | | | | | | | |

Table S2 Full result of MR estimates for the association between gut microbiota and adrenal disease

| Bacterial taxa (exposure) | MR method | No. of SNP | OR(95% CI) | P-value |
| --- | --- | --- | --- | --- |
| *Adrenocortical insufficiency* | | | | |
| Class. Deltaproteobacteria | IVW | 14 | 0.47(0.24,0.90) | 0.02 |
|  | MR Egger | 14 | 0.42(0.07,2.67) | 0.38 |
| Family. Desulfovibrionaceae | IVW | 12 | 0.43(0.21,0.87) | 0.02 |
|  | MR Egger | 12 | 0.47(0.07,3.18) | 0.46 |
| Family. Porphyromonadaceae | IVW | 11 | 4.12(1.72,9.85) | 0.001 |
|  | MR Egger | 11 | 2.89(0.05,1.74e2) | 0.62 |
| Genus. Lachnoclostridium | IVW | 15 | 2.25(1.12,4.52) | 0.02 |
|  | MR Egger | 15 | 3.81(0.35,4.19e1) | 0.29 |
| Order. Desulfovibrionales | IVW | 13 | 0.39(0.20,0.77) | 0.007 |
|  | MR Egger | 13 | 0.52(0.08,3.22) | 0.49 |
| Order. MollicutesRF9 | IVW | 16 | 1.73(1.03,2.92) | 0.04 |
|  | MR Egger | 16 | 2.47(0.48,1.28e1) | 0.30 |
| *Cushing syndrome* | | | | |
| Family. Acidaminococcaceae | IVW | 8 | 0.14(0.04,0.45) | 9.67e-4 |
|  | MR Egger | 8 | 0.23(0.01,6.36) | 0.42 |
| *Hyperaldosteronism* | | | | |
| Class. Methanobacteria | IVW | 12 | 0.58(0.39,0.89) | 0.01 |
|  | MR Egger | 12 | 0.87(0.18,4.35) | 0.87 |
| Family. Lactobacillaceae | IVW | 11 | 0.47(0.28,0.80) | 0.005 |
|  | MR Egger | 11 | 0.66(0.16,2.72) | 0.58 |
| Family. Methanobacteriaceae | IVW | 12 | 0.58(0.39,0.89) | 0.01 |
|  | MR Egger | 12 | 0.87(0.18,4.35) | 0.87 |
| Genus. Lactobacillus | IVW | 11 | 0.50(0.30,0.84) | 0.009 |
|  | MR Egger | 11 | 0.54(0.13,2.19) | 0.41 |
| Genus. Parasutterella | IVW | 16 | 2.05(1.17,3.58) | 0.01 |
|  | MR Egger | 16 | 2.10(0.41,1.85e1) | 0.39 |
| Genus. Peptococcus | IVW | 16 | 1.55(1.00,2.39) | 0.04 |
|  | MR Egger | 16 | 0.69(0.14,3.40) | 0.65 |
| Genus. Veillonella | IVW | 9 | 2.39(1.14,4.99) | 0.02 |
|  | MR Egger | 9 | 3.00e1(0.03,3.54e4) | 0.38 |
| Order. Methanobacteriales | IVW | 12 | 0.58(0.39,0.89) | 0.01 |
|  | MR Egger | 12 | 0.87(0.18,4.35) | 0.87 |
| MR, Mendelian randomization; SNP, single nucleotide polymorphism; OR, odds ratio; CI, confidence interval; IVW, inverse variance weighted. | | | | |

Table S3 The heterogeneity of gut microbiota instrumental variables.

| Bacterial taxa (exposure) | MR method | Q | Df | P-value |
| --- | --- | --- | --- | --- |
| *Adrenocortical insufficiency* | | | | |
| Class. Deltaproteobacteria | IVW | 12.88 | 13 | 0.46 |
|  | MR Egger | 12.87 | 12 | 0.38 |
| Family. Desulfovibrionaceae | IVW | 11.83 | 11 | 0.38 |
|  | MR Egger | 11.81 | 10 | 0.30 |
| Family. Porphyromonadaceae | IVW | 7.45 | 10 | 0.68 |
|  | MR Egger | 7.42 | 9 | 0.59 |
| Genus. Lachnoclostridium | IVW | 9.71 | 14 | 0.78 |
|  | MR Egger | 9.51 | 13 | 0.73 |
| Order. Desulfovibrionales | IVW | 12.38 | 12 | 0.42 |
|  | MR Egger | 12.27 | 11 | 0.34 |
| Order. MollicutesRF9 | IVW | 13.26 | 14 | 0.51 |
|  | MR Egger | 13.46 | 15 | 0.57 |
| *Cushing syndrome* | | | | |
| Family. Acidaminococcaceae | IVW | 5.89 | 7 | 0.55 |
|  | MR Egger | 5.79 | 6 | 0.45 |
| *Hyperaldosteronism* | | | | |
| Class. Methanobacteria | IVW | 2.54 | 11 | 0.99 |
|  | MR Egger | 2.28 | 10 | 0.99 |
| Family. Lactobacillaceae | IVW | 5.62 | 10 | 0.85 |
|  | MR Egger | 5.35 | 9 | 0.80 |
| Family. Methanobacteriaceae | IVW | 2.54 | 11 | 0.99 |
|  | MR Egger | 2.28 | 10 | 0.99 |
| Genus. Lactobacillus | IVW | 7.02 | 10 | 0.72 |
|  | MR Egger | 7.00 | 9 | 0.64 |
| Genus. Parasutterella | IVW | 13.14 | 15 | 0.59 |
|  | MR Egger | 13.14 | 14 | 0.52 |
| Genus. Peptococcus | IVW | 17.98 | 15 | 0.26 |
|  | MR Egger | 16.70 | 14 | 0.27 |
| Genus. Veillonella | IVW | 5.11 | 8 | 0.75 |
|  | MR Egger | 4.61 | 7 | 0.71 |
| Order. Methanobacteriales | IVW | 2.54 | 11 | 0.99 |
|  | MR Egger | 2.28 | 10 | 0.99 |
| MR, Mendelian randomization; IVW, inverse variance weighted. | | | | |

Table S4 Directional horizontal pleiotropy assessed by intercept term in MR Egger regression of the association between gut microbiota and adrenal disease

| Bacterial taxa (exposure) | Egger_intercept | SE | P-value |
| --- | --- | --- | --- |
| *Adrenocortical insufficiency* | | | |
| Class. Deltaproteobacteria | 0.01 | 0.07 | 0.91 |
| Family. Desulfovibrionaceae | -0.01 | 0.07 | 0.91 |
| Family. Porphyromonadaceae | 0.02 | 0.13 | 0.87 |
| Genus. Lachnoclostridium | -0.04 | 0.08 | 0.66 |
| Order. Desulfovibrionales | -0.02 | 0.07 | 0.76 |
| Order. MollicutesRF9 | -0.03 | 0.07 | 0.66 |
| *Cushing syndrome* | | | |
| Family. Acidaminococcaceae | -0.05 | 0.16 | 0.77 |
| *Hyperaldosteronism* | | | |
| Class. Methanobacteria | -0.07 | 0.13 | 0.62 |
| Family. Lactobacillaceae | -0.04 | 0.07 | 0.62 |
| Family. Methanobacteriaceae | -0.07 | 0.13 | 0.62 |
| Genus. Lactobacillus | -0.01 | 0.08 | 0.92 |
| Genus. Parasutterella | -0.01 | 0.07 | 0.97 |
| Genus. Peptococcus | 0.12 | 0.11 | 0.32 |
| Genus. Veillonella | -0.19 | 0.28 | 0.50 |
| Order. Methanobacteriales | -0.07 | 0.13 | 0.62 |
| MR, Mendelian randomization; SE, standard error. | | | |

Table S5 MR-PRESSO analysis for the association between gut microbiota and adrenal disease

| Bacterial taxa (exposure) | Method | Causal Estimate | SD | T | P-value | RSSobs | Global test P-value |
| --- | --- | --- | --- | --- | --- | --- | --- |
| *Adrenocortical insufficiency* | | | | | | | |
| Class. Deltaproteobacteria | MR-PRESSO | -0.76 | 0.33 | -2.30 | 0.04 | 14.75 | 0.51 |
| Family. Desulfovibrionaceae | MR-PRESSO | -0.85 | 0.36 | -2.35 | 0.04 | 13.87 | 0.43 |
| Family. Porphyromonadaceae | MR-PRESSO | 1.41 | 0.38 | 3.68 | 0.004 | 9.35 | 0.69 |
| Genus. Lachnoclostridium | MR-PRESSO | 0.81 | 0.30 | 2.73 | 0.02 | 10.98 | 0.80 |
| Order. Desulfovibrionales | MR-PRESSO | -0.93 | 0.34 | -2.70 | 0.02 | 14.41 | 0.47 |
| Order. MollicutesRF9 | MR-PRESSO | 0.54 | 0.25 | 2.17 | 0.05 | 15.46 | 0.58 |
| *Cushing syndrome* | | | | | | | |
| Family. Acidaminococcaceae | MR-PRESSO | -1.95 | 0.54 | -3.60 | 0.01 | 7.70 | 0.61 |
| *Hyperaldosteronism* | | | | | | | |
| Class. Methanobacteria | MR-PRESSO | -0.54 | 0.10 | -5.23 | 0.01 | 3.10 | 0.99 |
| Family. Lactobacillaceae | MR-PRESSO | -0.75 | 0.20 | -3.73 | 0.01 | 6.85 | 0.85 |
| Family. Methanobacteriaceae | MR-PRESSO | -0.54 | 0.10 | -5.23 | 0.01 | 3.10 | 0.99 |
| Genus. Lactobacillus | MR-PRESSO | -0.69 | 0.22 | -3.13 | 0.01 | 8.47 | 0.74 |
| Genus. Parasutterella | MR-PRESSO | 0.72 | 0.27 | 2.68 | 0.02 | 14.88 | 0.60 |
| Genus. Peptococcus | MR-PRESSO | 0.44 | 0.22 | 1.97 | 0.07 | 20.15 | 0.30 |
| Genus. Veillonella | MR-PRESSO | 0.87 | 0.30 | 2.90 | 0.02 | 6.44 | 0.77 |
| Order. Methanobacteriales | MR-PRESSO | -0.54 | 0.10 | -5.23 | 0.01 | 3.10 | 0.99 |
| MR, Mendelian randomization; SD, standard deviation; RSSobs, observed residual sum of squares. | | | | | | | |

Table S6 Full result of MR estimates for the association between adrenal disease and gut microbiota

| Bacterial taxa (exposure) | MR method | OR(95% CI) | P-value | Egger intercept | SE | P-value |
| --- | --- | --- | --- | --- | --- | --- |
| *Adrenocortical insufficiency* | | | | | | |
| Class. Deltaproteobacteria | IVW | 0.98(0.95,1.00) | 0.06 | 0.01 | 0.02 | 0.67 |
|  | MR Egger | 0.95(0.84,1.07) | 0.44 |  |  |  |
| Family. Desulfovibrionaceae | IVW | 0.98(0.95,1.00) | 0.07 | 0.01 | 0.02 | 0.62 |
|  | MR Egger | 0.95(0.84,1.07) | 0.41 |  |  |  |
| Family. Porphyromonadaceae | IVW | 1.01(0.98,1.03) | 0.54 | -0.003 | 0.02 | 0.90 |
|  | MR Egger | 1.01(0.90,1.14) | 0.81 |  |  |  |
| Genus. Lachnoclostridium | IVW | 1.00(0.97,1.02) | 0.71 | 0.005 | 0.03 | 0.87 |
|  | MR Egger | 0.98(0.86,1.12) | 0.81 |  |  |  |
| Order. Desulfovibrionales | IVW | 0.98(0.95,1.00) | 0.08 | 0.01 | 0.02 | 0.62 |
|  | MR Egger | 0.95(0.84,1.07) | 0.41 |  |  |  |
| Order. MollicutesRF9 | IVW | 1.00(0.97,1.03) | 0.88 | 0.05 | 0.03 | 0.12 |
|  | MR Egger | 0.88(0.76,1.02) | 0.14 |  |  |  |
| *Cushing syndrome* | | | | | | |
| Family. Acidaminococcaceae | IVW | 0.99(0.97,1.01) | 0.21 | -0.03 | 0.02 | 0.26 |
|  | MR Egger | 1.03(0.97,1.09) | 0.41 |  |  |  |
| *Hyperaldosteronism* | | | | | | |
| Class. Methanobacteria | IVW | 1.02(0.98,1.06) | 0.38 | 0.01 | 0.05 | 0.86 |
|  | MR Egger | 1.00(0.82,1.23) | 0.99 |  |  |  |
| Family. Lactobacillaceae | IVW | 1.02(0.99,1.05) | 0.14 | -0.01 | 0.02 | 0.57 |
|  | MR Egger | 1.05(0.95,1.16) | 0.36 |  |  |  |
| Family. Methanobacteriaceae | IVW | 1.02(0.98,1.06) | 0.38 | 0.01 | 0.05 | 0.86 |
|  | MR Egger | 1.00(0.82,1.23) | 0.99 |  |  |  |
| Genus. Lactobacillus | IVW | 1.02(0.99,1.05) | 0.15 | -0.02 | 0.02 | 0.35 |
|  | MR Egger | 1.07(0.97,1.19) | 0.22 |  |  |  |
| Genus. Parasutterella | IVW | 1.01(0.99,1.04) | 0.26 | 0.01 | 0.02 | 0.76 |
|  | MR Egger | 1.00(0.92,1.09) | 0.99 |  |  |  |
| Genus. Peptococcus | IVW | 1.01(0.98,1.04) | 0.51 | 0.02 | 0.03 | 0.48 |
|  | MR Egger | 0.97(0.86,1.09) | 0.61 |  |  |  |
| Genus. Veillonella | IVW | 1.00(0.97,1.04) | 0.90 | -0.03 | 0.03 | 0.25 |
|  | MR Egger | 1.08(0.96,1.21) | 0.25 |  |  |  |
| Order. Methanobacteriales | IVW | 1.02(0.98,1.06) | 0.38 | 0.01 | 0.05 | 0.86 |
|  | MR Egger | 1.00(0.82,1.23) | 0.99 |  |  |  |
| MR, Mendelian randomization; OR, odds ratio; CI, confidence interval; IVW, inverse variance weighted; SE, standard error. | | | | | | |
